# Developing and assessing MyTBCompanion – A tri-lingual integrated video observed therapy app for tuberculosis patient management in Malaysia and Indonesia

**DOI:** 10.1371/journal.pone.0320394

**Published:** 2025-04-29

**Authors:** Vimala Balakrishnan, Thayananth Kumaresan, Henry Surendra, Matthew Allsop, Chiong Kian Tiong, Nicholas Ng Jian Sheng, Mazlina Mohtar

**Affiliations:** 1 Faculty of Computer Science and Information Technology, Universiti Malaya, Kuala Lumpur, Malaysia; 2 Department of Computer Science and Engineering, Korea University, Seoul, Korea; 3 Monash University Indonesia, Tangerang, Banten, Indonesia; 4 Oxford University Clinical Research Unit Indonesia, Faculty of Medicine, Universitas Indonesia, Jakarta, Indonesia; 5 Faculty of Medicine and Health, Leeds Institute of Health Sciences, University of Leeds, Leeds, United Kingdom; 6 Faculty of Medicine, Universiti Malaya, Kuala Lumpur, Malaysia; 7 Malaysian Association for the Prevention of Tuberculosis, Kuala Lumpur, Malaysia; Universiti Kebangsaan Malaysia Faculty of Medicine: Hospital Canselor Tuanku Muhriz UKM, MALAYSIA

## Abstract

**Background:**

Limited studies have developed a mobile phone-based application that supports Asynchronous Video Observed Therapy (A-VOT) for Tuberculosis (TB) program. This study aimed to design, develop, and assess MyTBCompanion, a mobile phone-based digital health intervention to support A-VOT for TB management and care among low-income patients in Malaysia and Indonesia.

**Methods:**

MyTBCompanion was designed and developed under a partnership involving experts in information technology, respiratory disease, and linguistics. Pre and Post-test surveys were done to assess feedback on the existing TB strategies (Direct Observed Therapy (DOT) and/or VOT) and the usability of the tested A-VOT strategy using MyTBCompanion. We collected data on the patient’s age, education level, current treatment strategy, and statements measuring four MyTBCompanion components: Engagement (how interesting, customizable, interactive and well-targeted to an audience an app is), Functionality (ease of use, app design, navigation), Aesthetic (the graphic design, overall visual appeal, color scheme, and stylistic consistency) and Information (content accuracy and relevance). The Mann-Whitney U test was performed for group comparisons, with results considered significant at p < 0.05.

**Results:**

In total, 49 patients with TB were recruited from Malaysia (n = 29) and Indonesia (n = 20). Most participants in both countries were 20–40 years old. Indonesian participants mainly had tertiary education (11/20, 55.0%), whereas most had secondary education level in Malaysia (17/29, 58.6%). Most Malaysian participants (19/29, 65.5%) were using VOT through WhatsApp, with fewer (10/29, 34.5%) using DOT. All participants from Indonesia (20/20, 100%) were using the DOT strategy. Overall, compared to the existing strategies, a higher mean agreement score was observed for MyTBCompanion, with Information scoring the highest agreement (4.57/5.0), followed by Engagement (4.53/5.0), Functionality (4.51/5.0) and Aesthetic (4.49/5.0).

**Conclusion:**

Findings suggest an overall good agreement on the usability of A-VOT strategy using MyTBCompanion in terms of engagement, functionality, information and aesthetics, with many indicating their willingness to recommend it to others, marking an encouraging milestone in the app’s development.

## Introduction

Tuberculosis (TB) remains a significant global health challenge (an estimated 10.6 million cases in 2021), with almost 45% of cases being from Southeast Asia (SEA) [1]. The World Health Organization (WHO) estimated 92 cases per 100,000 population in 2019 for Malaysia, with an upward trajectory over recent years. A similar upward trajectory is noted in Indonesia, as the average cases increased from 443,235 in 2021–717,941 in 2022, placing Indonesia as the second highest TB burden country in the world [1]. Recent evidence from Indonesia suggests that the COVID-19 pandemic has led to a dramatic decrease in notified TB cases and a significant reduction in treatment coverage, partly related to interrupted or scaled-down TB services and changes in patient health-seeking behaviors during the early pandemic phase [2].

WHO endorsed the Direct Observed Treatment Short-Course (DOTS or DOT) therapy as part of the TB treatment regime and is currently practised in most low- and middle-income countries, including within SEA. DOTS necessitates patients to physically visit healthcare facilities for their prescribed medications, typically spanning a duration of 4–6 months, or possibly more. During the initial two months of treatment, DOTS staff or assigned treatment observers vigilantly oversee the ingestion of medications, a pivotal period that extends until the patient reaches a non-infectious state. However, the implementation of this strategy poses formidable challenges to the patients, caregivers as well as healthcare practitioners [3,4] including a range of societal and geographical factors such as stigma, poverty, logistical constraints related to travel, and limited healthcare accessibility [5–7].

Mobile health (mHealth) platforms including apps have brought numerous benefits to the healthcare industry and individuals alike. However, development and implementation of apps to support TB and its management is lacking. The use of an mHealth app for TB signifies a shift from DOT to Video Observed Therapy (VOT), either synchronously (S-VOT) (i.e., both patients and healthcare online at the same time) or asynchronously (A-VOT) [8,9]. Most of the studies on A-VOT were conducted in Western countries [8,9], and only one was found in Vietnam [10]. However, all studies examine the feasibility of A-VOT adoption using video uploading services via existing apps such as WhatsApp or Telegram rather than bespoke mobile phone apps. Moreover, WhatsApp and Telegram, for example are not suitable for VOT in TB treatment due to their lack of compliance features, inadequate privacy protections for sensitive health data, and inability to integrate with healthcare systems for monitoring patient progress. Further, they do not offer tailored content, structured feedback mechanisms, or analytical tools to track treatment effectiveness, hence limiting their effectiveness in supporting TB patient management [11]. Therefore, a dedicated, integrated TB app is essential, especially for low-income populations, as it can provide tailored support and resources to effectively address their unique needs in managing the disease. Nevertheless, these studies reported their respondents to be receptive to A-VOT. For instance, US-based studies have reported A-VOT users to have a significantly higher adherence compared to DOT, with the majority willing to recommend the method to others [9,12,13]. A similar finding was reported by Nguyen and colleagues in Vietnam as well [10].

To the best of our knowledge, most of these studies were pilot studies with A-VOT implemented using existing video-recording tools to assess its impact on TB treatment adherence. Except for a study in Argentina [14] using an integrated mHealth for TB testing, no studies have developed a mobile phone-based application that supports A-VOT for the TB community. Notably, there has been no research to date exploring the use of mHealth approaches for TB treatment management in the specific contexts of Indonesia and Malaysia. To ensure the development of scalable and acceptable digital solutions for TB, it is crucial to develop and test localized solutions in the context in which they are intended for use [15]. To address this gap and explore the role of A-VOT for TB in low- and middle-income country settings, a TB-centric mHealth app integrated with A-VOT (herein referred to as MyTBCompanion) as well as other functions was developed to improve treatment adherence among the low-income TB patients. The present study reports on the development and usability assessment of MyTBCompanion among low-income TB patients from two SEA countries: Malaysia and Indonesia. Other notable key functions of the app include side-effect reporting, teleconsultation appointment booking, educational materials and a chatbot.

## Materials and methods

**Fig 1:**
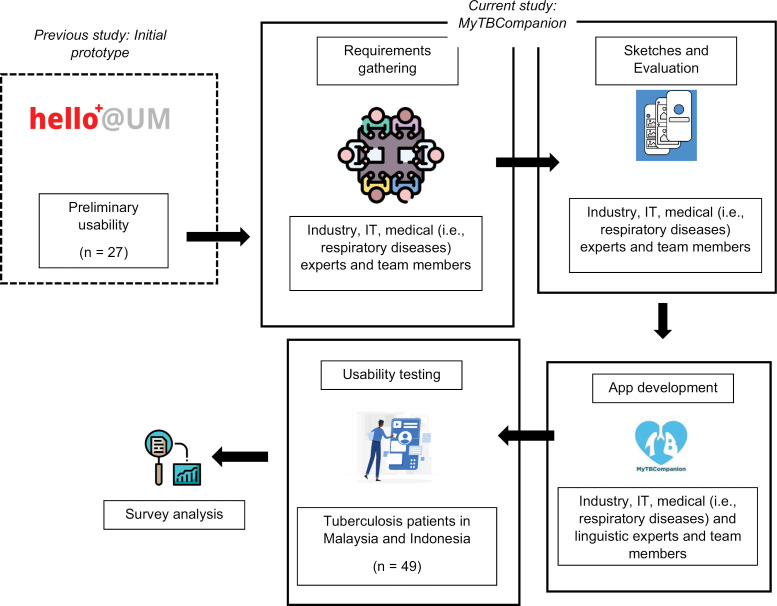
MyTBCompanion design and development process.

Fig 1 depicts the process flow for the design and development of the mobile phone app, MyTBCompanion, developed through an academic partnership with an industry specializing in app development.

The initial prototype (known as Hello@UM) for MyTBCompanion was developed following an iterative cycle whereby low-fidelity outcomes were used to iteratively inform the design of high-fidelity prototypes – an approach that is widely adopted in prototyping and software development projects [14]. This approach involves creating and refining design concepts through repeated testing and feedback. Initially, low-fidelity prototypes, such as sketches or wireframes, are developed to visualize the app’s basic functionality and layout, allowing for quick adjustments based on user feedback. After validating core ideas, high-fidelity prototypes are developed to closely resemble the final product and undergo further user testing. This cyclical process ensures the app is refined based on user needs, resulting in a more effective and user-friendly final product [14]. The initial prototype was developed as part of another study. The feedback solicited from the preliminary usability study based on the prototype was used as input to the initial phase of the current study as shown in **Fig 1**. The feedback was solicited through discussions and brief surveys involving 27 individuals comprising TB medical experts (n = 2) and students (n = 5), IT experts (n = 7) and post-graduate students (n = 13). The feedback was generally positive, with a few suggestions for improvement. We adopted the same iterative cycle in the design and development of MyTBCompanion, and these are elaborated subsequently.

### Requirements gathering

The development of MyTBCompanion had undergone initial development through consultation with domain experts comprising respiratory diseases, IT experts and industry. During the requirements gathering phase, the comments and findings from the preliminary usability analysis were discussed, and additional features were identified for incorporation. These specifically included refining the graphical user interfaces (GUI) in terms of layout and color (e.g., blue was selected as the main theme as the color is deemed to promote a tranquil environment [16,17], improving screen navigation, and visuals, among others. Table 1 illustrates the relationship between the feedback collected, the changes implemented, and the evaluation dimensions assessed in this study (refer to Assessments for further details).

**Table 1:** Mapping of feedback/comment to dimensions.

Stage	Feedback	Dimension	Changes
Preliminary usability analysis – previous study	More or other colors	Aesthetic	Original theme of red changed to blue
	Support Malay language	Functionality	Support three languages – English, Malay and Indonesian
Requirement gathering	Increase icon and font sizes for some of the screens	Functionality	Increased accordingly
	Improve some of the screen flow	Functionality	Changed to ensure a seamless flow between the screens
	Include a visual progress tracker for patient	Engagement	A gauge-like tracker incorporated for patients and healthcare officers

### Sketches and evaluation

High-fidelity digital wireframes were created using Figma, a collaborative interface design tool, to establish the layout and functionality. Bi-weekly meetings were held with the IT industry developer (i.e., n = 3 experts). Points or issues specifically pertaining to medical terms/processes were communicated with the respiratory diseases’ experts for clarification and verification. These brainstorming and assessment processes were iteratively undertaken through multiple cycles until a consensus was achieved. This stage consumed approximately three weeks in total.

### App development

In this stage, the industry experts converted the high-fidelity frames into a fully functioning mobile phone app using React Native, a popular JavaScript-based mobile app framework that enables the development of cross-platform apps (i.e., iOS and Android). The main researcher, including the IT experts from the team, had bi-weekly meetings to monitor the progress of MyTBCompanion development. During these meetings, ideas were exchanged to improve designs and navigations, and logical errors were highlighted and corrected. The medical experts joined the meetings towards the end of the development and assessed the app, particularly the process flow. MyTBCompanion was initially developed in English. Upon its completion, the Google API translator was embedded to support two other languages, namely, Malay (i.e., the national language of Malaysia) and Indonesian languages. Two linguistic experts from each country checked and verified the translations.

MyTBCompanion provides two user roles: patient and healthcare provider. The present study primarily focuses on the patient module and its key features, detailed below.

#### Patient - A-VOT support.

**Fig 2:**
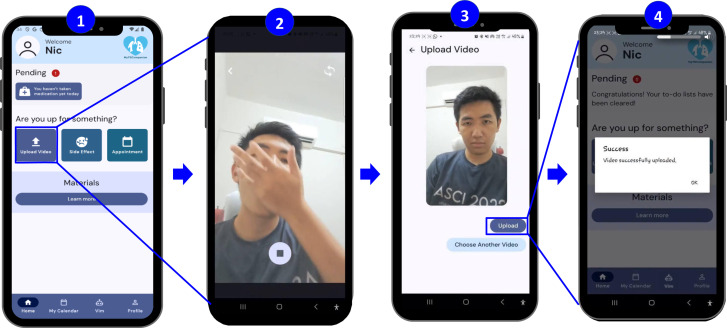
Asynchronous video-observed therapy support.

**Fig 2** illustrates the screen navigation for A-VOT. Screen #1 appears upon patient registration (first-time user) and login, displaying a to-do list and three key functions: video upload, side-effect reporting, and teleconsultation appointment booking. A patient clicks on the video upload button and records a video of themselves self-medicating (#2). Upon uploading (#3), an acknowledgement will be displayed (#4). DOT staff can review these videos at their convenience using the healthcare provider module. In instances where a patient neglects to submit a video, a healthcare provider will initiate contact with the patient through a phone call.

#### Patient - Side-effect reporting.

**Fig 3:**
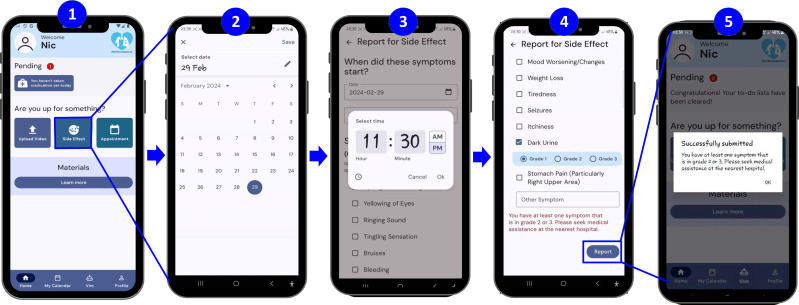
Side-effect reporting.

The second key function is side-effect reporting (**Fig 3**), whereby TB patients can report any side-effects experienced using the app, which will be reviewed by the DOT staff and/or healthcare professionals for appropriate action (e.g., consultation, phone call, etc.) (**Fig 3**). This is done by clicking on the side-effect reporting button (#1) and specifying the date (#2) and time (#3) of symptom onset. A list of standard TB-related side effects (including an ‘Others’ option) is presented for patients to choose from (#4). These side effects were identified through a literature review and verified by the TB experts supporting the development of the app. Side effects are categorized into three grades: Grade 1, 2, and 3 (with an alert prompting patients to seek medical assistance for Grades 2 and 3). The final screen (#5) confirms successful submission.

Other functions for patients include making a teleconsultation appointment with the healthcare provider for consultancy matters, a personalized progress tracker that provides visuals on the patient’s progress in self-medicating, and a chatbot, Vim®, that can handle textual communication. Additionally, a comprehensive repository of Frequently Asked Questions (FAQs) is accessible and presented in diverse formats, including text, images, and videos. For direct engagement, the option to contact nurses through telephone calls is also readily available (complete screen snaps are available in S1 Appendix).

**Fig 4:**
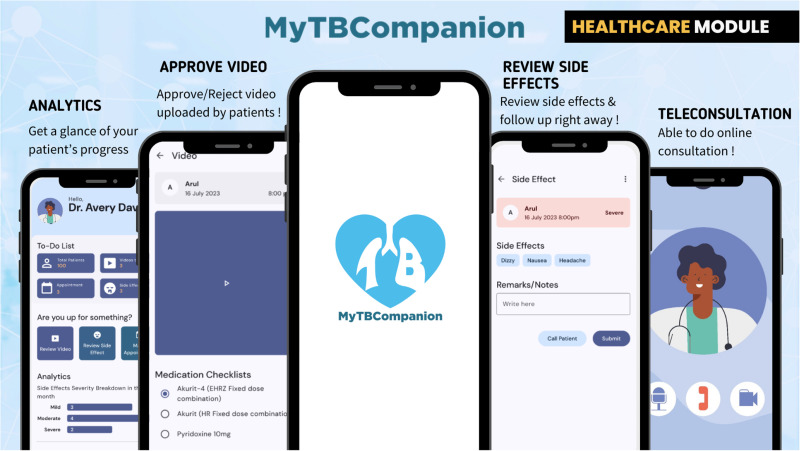
Healthcare key functions (Dr Avery icon designed by Freepik).

The functions of the healthcare or DOT staff are primarily to review the uploaded videos (approve/reject), review the side effects, provide tele-consultation, and monitor patients’ progress. **Fig 4** depicts the basic screens for these functions.

### Assessments

#### Instrument.

Pre- and post-test questionnaire surveys were prepared in English for the usability test. The survey questionnaires were structured into four sections, with minor adjustments made for the post-test version. The pre-test questionnaire included: Section I, which collected demographic details (age, education level, current treatment strategy, treatment duration etc.); Sections II and III, which gathered feedback on patients’ current treatment strategies, that is, DOTS or VOT, respectively. Section III was adapted from the Mobile Application Rating Scale (MARS) [18] and mHealth App Usability Questionnaire (MAUQ) [19] comprising five dimensions in total. Only items relevant to assessing app usability were adapted. For the pre-test, Section III contained 10 statements focusing on three dimensions, namely Engagement (how interesting, customizable, interactive and well-targeted to the audience an app is), Functionality (ease of use, app design, navigation etc.), and Aesthetic (the graphic design, overall visual appeal, color scheme, and stylistic consistency). The fourth dimension, Information (accuracy and relevance of content) was excluded as it was unsupported by the existing apps (e.g., WhatsApp). Participants currently using other apps for VOT rated their agreement/disagreement with each statement on a five-point Likert scale (adapted from Section F in MARS [18]). Considering these were measured using a Likert-scale, the mean scores are provided for all the items for each of these dimensions. Finally, the fifth dimension, Subjective Quality was included in Section IV, asking participants about their willingness to recommend the current strategy (yes, maybe, no) and providing an overall star rating (out of five), along with a space for additional comments (if any). All the statements in Sections III and IV were adapted to suit our study (e.g., the word app replaced with MyTBCompanion). Both the MARS and MAUQ tools have been validated in previous studies [14,18,19], establishing the reliability of our adapted pre- and post-test surveys. This study aligns with the approach used by authors in [14], who assessed the usability of a TB diagnosis mobile app using MARS, further reinforcing the validity of our chosen instruments.

For the post-test questionnaire, Section I was simplified to only require participants to state the language used for the app and Section II was removed. Section III was expanded to include Information, and the Subjective Quality component was updated to include a question on participants’ willingness to pay for the app (yes, maybe, no). The post-test questionnaire was utilized by participants to evaluate MyTBCompanion. It was originally developed in English and then translated and verified by linguistic experts for Malay and Indonesian. The complete questionnaires can be found in the S2 Appendix.

#### Procedure.

The usability tests were conducted in Malaysia and Indonesia. Specifically, low-income TB patients were identified with the help of the Malaysian Association for Prevention of Tuberculosis (i.e., a local NGO) encompassing three states, that is, Selangor, Kuala Lumpur and Pahang. Both Selangor and Kuala Lumpur are developed urban regions with a high TB prevalence, whereas Pahang is deemed to be a less-developed state [20]. For the Indonesian team, the patients were recruited from a public hospital working under the coordination of the Jakarta Provincial Health Office. Both these entities continuously work with clinics providing TB treatment and care to the patients; hence, they had access to patient records. In Malaysia, the low-income population (a.k.a. B40 representing the bottom 40% of income earners) refers to individuals with household income less than 5,250 MYR (1,098 USD). A similar categorization is applicable in Indonesia where those with household income less than 1,035 USD are considered as low-income population. This took place between April 1, 2024, and April 30, 2024.

The survey questionnaires were printed and distributed to TB centers in Selangor and Kuala Lumpur, while a Google Form link was shared with teams in Pahang and Indonesia due to geographical constraints. Additionally, the link to access MyTBCompanion was provided as part of the usability test. To ensure smooth data collection, researchers prepared instructional videos in both English and Malay, conducted demonstration sessions (both online and in-person) to showcase the functionality of MyTBCompanion, and guided TB center representatives through the questionnaires. Four researchers were involved, each assigned to a specific location. These representatives, along with healthcare officers (i.e., nurses) at the TB clinics, facilitated the usability test.

TB patients who fulfilled the study criteria (i.e., adults currently diagnosed with TB and undergoing treatment) were approached by the healthcare officers to participate in the study during their daily treatment visits. As patients were from various regions, each TB center in Malaysia was responsible for recruiting ten patients to ensure similar sample size for a fair comparison analysis. Similarly, twenty participants were targeted in Indonesia as recruitment was done by a single TB center. Generally, all those approached were willing to participate in the study except for one respondent in Malaysia, hence a high response rate is noted (i.e., 98%). This resulted in a total of 49 TB patients (Malaysia = 29; Indonesia = 20), a figure that is deemed substantial for usability studies as shown in previous research [14].

**Fig 5:**
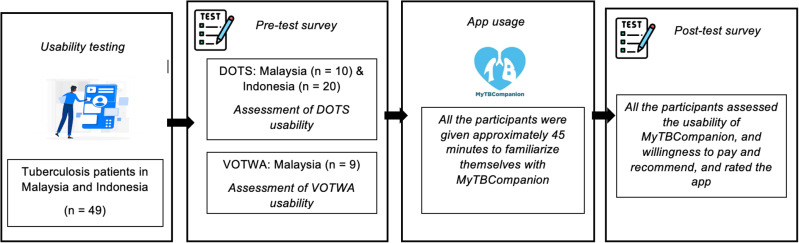
Usability testing process flow.

Recruited patients who fulfilled the criteria had to provide their consent before the data collection (i.e., written consent through the questionnaire). Patient participants were briefed on the study, app content and features and asked to install the app on their phones. Patients had to answer the pre-test questionnaire prior to using MyTBCompanion. They had to perform three key tasks: upload a video, report any side effects and book a teleconsultation appointment. Approximately 45 minutes were given for participants to explore the app on their own (guided by the healthcare officers for elderly patients, if needed) before answering the post-test questionnaire. A summary of the key steps outlining the usability testing phase is illustrated in **Fig 5** below. All participants received a token of appreciation after completing the post-test questionnaire.

### Data analysis

The Statistical Package for Social Sciences (SPSS) version 28 was used for the data analysis. Mann-Whitney U test was performed for group comparisons as some of the group sizes were small (i.e., < 15), with results considered significant at p < 0.05. The letters M and SD are used to denote mean and standard deviation, respectively. The Malaysian sample was segregated into two: DOT versus VOTWA (i.e., VOT using WhatsApp) in some of the analyses.

### Ethics approval

This study was approved by the Universiti Malaya Research Ethics Committee (UM.TNC2/UMREC_2896) and Health Research Ethics Committee of Universitas Negeri Semarang (135/KEPK/FK/KLE/2024). Written individual informed consent was obtained from all participants. The individual pictured in Fig 2 and S1 has provided written consent (as outlined in the PLOS consent form) to publish their image alongside the manuscript. All the data collection and analysis method used in this study complied with the terms and conditions for the source of the data.

## Results

### General characteristics of participants

A total of 49 TB patients were recruited for the study, with 29 participants from Malaysia and 20 from Indonesia. In Malaysia, participants were recruited from Selangor (n = 9), Pahang (n = 10), and Kuala Lumpur (n = 10), while all participants from Indonesia (n = 20) were based in Jakarta. **Table 2** provides the demographic details of all the participants. Most participants (31/49;63.2%) were 20–40 years old. Indonesian participants mainly had tertiary education (n = 11/20;55.0%), whereas most had secondary education level in Malaysia (n = 17/29;58.6%). Similar patterns were observed for the duration of years using mobile phones, with both Malaysia (n = 14/29;48.3%) and Indonesia (n = 8/20;40.0%) recording the highest for the 6–15 years category.

**Table 2:** Demographic profiles of the study participants.

Variables	Categories	Total by country		By region for Malaysia		
		Malaysia	Indonesia	Pahang	Selangor	KL
		*n (%)*	*n (%)*	*n (%)*	*n (%)*	*n (%)*
Age (years)	20–40	17 (58.6)	14 (70.0)	4 (40.0)	5 (55.6)	8 (80.0)
	41–60	8 (27.5)	6 (30.0)	3 (30.0)	3 (33.3)	2 (20.0)
	More than 60	4 (13.7)	0 (0.0)	3 (30.0)	1 (11.1)	0 (0.0)
Education level	Primary	4 (13.7)	6 (30.0)	2 (20.0)	1 (11.1)	1 (10.0)
	Secondary	17 (58.6)	3(15.0)	7 (70.0)	8 (88.9)	2 (20.0)
	Tertiary	8 (27.5)	11(55.0)	1 (10.0)	0 (0.0)	7 (70.0)
Mobile phone experience (years)	Less than 5	5 (17.2)	6 (30.0)	2 (20.0)	3 (33.3)	0 (0.0)
	6–15	14 (48.3)	8 (40.0)	6 (60.0)	4 (44.4)	4 (40.0)
	More than 16	10 (35.0)	6 (30.0)	2 (20.0)	2 (22.2)	6 (60.0)
VOT awareness	Yes	26 (89.7)	1 (05.0)	7 (70.0)	9 (100)	10 (10.0)
	No	3 (10.3)	19 (95.0)	3 (30.0)	0 (0.0)	0 (0.0)
Current strategy	DOT	10 (35.0)	20 (100)	10 (100.0)	0 (0.0)	0 (0.0)
	VOT (WhatsApp)	19 (65.5)	0 (0.0)	0 (0.0)	9 (100)	10 (10.0)
Treatment duration (months)	Less than one	6 (20.7)	2 (15.0)	1 (10.0)	3 (33.3)	2 (20.0)
	Between 1–2	10 (35.0)	3 (10.0)	4 (40.0)	2 (22.2)	4 (40.0)
	More than 2	13 (44.8)	15 (75.0)	5 (50.0)	4 (44.4)	4 (40.0)

VOT awareness is very low among participants from Indonesia, with most (19/20, 95%) reporting no awareness. This is in contrast with participants from Malaysia, where the majority (26/29, 89.7%) reported being aware of VOT. This aligns with the treatment strategies implemented in which some participants from Malaysia (19/29, 65.5%) were using VOT through WhatsApp, with fewer (10/29, 34.5%) using DOT. All participants from Indonesia (20/20, 100%) were using the DOT strategy. Most participants had been on their treatment for more than two months in Malaysia (13/29, 44.8%) and Indonesia (15/20, 75.0%), followed by those between one and two months in Malaysia (10/29, 34.5%) and Indonesia (3/20, 15.0%).

### Pre-test results

The overall mean score for agreement towards the current strategy employed was found to be similar between the countries, particularly for DOT (i.e., Malaysia_DOT_ = 4.24/5.0; Indonesia_DOT_ = 4.29/5.0). However, the agreement mean score for Malaysians using VOTWA was higher, that is, 4.43/5.0. Specifically, the mean score for each component was: Engagement = 4.43/5.0, Functionality = 4.51/5.0 and Aesthetic = 4.37/5.0. As for the star rating for the current treatment strategy, all Malaysian patients gave a five-star for DOT, unlike the Indonesian counterpart (i.e., M = 4.45/5.0). The mean rating for VOTWA was lower, that is, 4.37/5.0. Qualitative feedback was not many, of these, one Malaysian and two Indonesians stated their preferences for DOT as they prefer communicating directly with the nurses. Three Malaysians claimed parking difficulties. One VOTWA participant commented on the need to include more TB-related information pictures for the current strategy.

### Post-test results

Table 3 shows the analysis results between the DOT patients in both countries, with stark differences between agreement scores. Participants in Malaysia scored a higher level of disagreement for all four components. Mann-Whitney U test results show all the differences to be significant (i.e., p < 0.001).

**Table 3:** DOT patients’ perceptions towards MyTBCompanion in Malaysia and Indonesia.

Factors	Malaysia (n = 10) M(SD)	Indonesia (n = 20) M(SD)	p-value
Engagement	2.68 (0.82)	4.36 (0.45)	**< 0.001**
Functionality	2.74 (0.87)	4.33 (0.55)	**< 0.001**
Aesthetics	2.60 (0.94)	4.25 (0.44)	**< 0.001**
Information	2.83 (0.62)	4.40 (0.41)	**< 0.001**
Average	2.71 (0.81)	4.34 (0.46)	–

Note: M: mean; SD: standard deviation; p-value significant at p < 0.05

A comparison between VOTWA and MyTBCompanion among the remaining 19 participants in Malaysia indicated a higher agreement for MyTBCompanion, albeit not reaching statistical significance at conventional levels (Table 4). Nevertheless, the differences ranged between 0.01 (Functionality) and 0.12 (Aesthetic), with preferences towards MyTBCompanion. The overall mean score was marginally higher between pre-test (M = 4.43/5.0) and post-test (M = 4.48/5.0) scores.

**Table 4:** VOTWA versus MyTBCompanion in Malaysia.

Malaysia (n = 19)	Engagement	Functionality	Aesthetics	Information	Average
	M (SD)				
Pre-test	4.43 (0.82)	4.51 (0.78)	4.37 (0.72)	–	4.43 (0.77)
Post-test	4.53 (0.70)	4.52 (0.62)	4.49 (0.69)	4.57 (0.55)	4.48 (0.64)

Note: Information was excluded from the pre-test survey.

### Subjective quality assessment

Results were positive for both Malaysia (19/29;55.1%) and Indonesia (19/20;95.0%), as most of the patients were willing to recommend MyTBCompanion to others, regardless of the current treatment strategy used (see Table 5). This was followed by those who were unsure (i.e., Maybe) in both countries. In both countries, most participants were unwilling to pay to use MyTBCompanion, including Malaysia (14/29;48.2%) and Indonesia (10/20;50.0%). The average star rating for MyTBCompanion was very low among Malaysian DOT patients (i.e., M = 2.00; SD = 0.94). However, higher ratings were observed among the Indonesian DOT patients (M = 4.35; SD = 0.81) and Malaysian VOTWA patients (M = 4.47; SD = 0.61), indicating a generally satisfactory rating among these participants.

**Table 5:** Subjective quality assessment between Malaysia and Indonesia.

	Categories	Pre-test		Post-test	
		Malaysia	Indonesia	Malaysia	Indonesia
Recommend (Frequency)	Yes	25 (86.20)	16 (80.00)	16 (55.10)	19 (95.00)
	No	0 (0.00)	1 (5.00)	6 (20.70)	0 (0.00)
	Maybe	4 (13.80)	3 (15.00)	7 (24.20)	1 (5.00)
Willing to Pay (Frequency)	Yes	NA	NA	5 (17.30)	3 (15.00)
	No	NA	NA	14 (48.20)	10 (50.00)
	Maybe	NA	NA	10 (34.50)	7 (35.00)
Rating M (SD)	DOT	5.00 (0.00)	4.45 (0.89)	2.00 (0.94)	4.35 (0.81)
	VOTWA	4.37 (0.78)	NA	4.47 (0.61)	NA

### Post-test qualitative feedback

Limited qualitative feedback was provided by participants through the optional comment in Section IV of the post-test questionnaire. Participants (n = 5) stated a preference for meeting health professionals face-to-face. Individual feedback from a VOTWA participant included satisfaction with the application, and a Malaysian DOT patient commented on their lack of technology skill in using MyTBCompanion. An individual Indonesian DOT patient expressed hope for the MyTBCompanion app accessibility to all, another wished for it to be available for free initially, and another hoped for its suitability for elderly users.

## Discussion

This study describes the development and usability of MyTBCompanion, an integrated mobile phone app embedded with the A-VOT technology to improve TB treatment adherence in Malaysia and Indonesia. Pre- and post-test survey findings indicate mixed responses among the DOT and VOT patients in Malaysia towards MyTBCompanion. In the pre-test phase, we found there were high levels of agreement across existing treatment strategies (DOT and VOTWA) that participants were accessing. However, comparisons with existing treatment strategies and the intervention, MyTBCompanion, highlighted key differences based on treatment strategy. For patients receiving DOT in Indonesia and VOTWA (in Malaysia only), MyTBCompanion was comparable across ratings relating to Engagement, Functionality, Aesthetics, and Information. However, for patients in Malaysia receiving DOT, MyTBCompanion was rated significantly lower across all factors.

The development of digital health approaches to support treatment adherence for TB is emerging, although with variable effects [21–23]. Evidence is promising for their use with, for example, mobile phone-based approaches for TB, having also been found to support wider skills and confidence development that may translate to digital health engagement for a wider range of conditions [23,24]. This study contributes potential insights into factors that may influence interaction with and use of mobile phone applications for TB management. Across all post-test scores, Information was rated highly, indicating that the content used in MyTBCompanion was reliable and relevant to users.

Engagement, too, received high scores. This may have been attributable to supplementary functions embedded in the app, such as Vim, a chatbot that enables a user to raise basic TB-related questions alongside contact numbers to DOT staff (i.e., a single click will initiate the phone call). Additionally, the comprehensive Frequently Asked Questions (FAQs) comprising content in various formats provide an easy and interesting mechanism for an individual to educate himself on TB-related matters, thus improving their health literacy. Similarly, patients and/or caregivers can monitor the daily medication progress through the color-coded calendar that uses distinct colors to represent medication status, hence video submission status (e.g., green to indicate submitted, red to indicate submission failure, etc.). This not only provides TB patients with a clear and intuitive way to monitor and manage their medication schedule, but it may also provide a sense of accomplishment as they see a pattern of completed doses (i.e., progress score in percentage).

Results also indicate high agreement for Functionality, probably attributable to the app design itself, where simplicity was emphasized, acknowledging the diverse backgrounds of the intended users, including factors such as their education levels and familiarity with technology. Further, the app enables a seamless navigation experience. Hence, users are not overloaded or confused in accomplishing an intended task. This aligns with previous studies that have shown the importance of mobile apps to provide good navigation to their users [25].

The Subjective component revealed most TB patients are willing to recommend MyTBCompanion to others, regardless of the current treatment strategy used. This is particularly true for Indonesian TB patients (95%) who are currently on DOT strategy and were not aware of VOT. However, it should be noted that most of the TB patients in both countries stated that they would be unwilling to pay to use the app, indicating the need of additional programmatic efforts to make the app freely available for patients. The very low average star rating for MyTBCompanion among Malaysian DOT patients might be linked to a lack of technology skills in using MyTBCompanion app and a personal preference to meet health practitioners in person, as suggested by the qualitative feedback. It is also to note that all the Malaysian DOT patients were recruited from Pahang, a lesser-developed state, compared to the urbanized participants from Kuala Lumpur and Selangor. The higher average star rating among the Indonesian DOT patients might be due to the younger age distribution and higher education level of the patients compared to those in Malaysia. For comparison, the proportion of patients aged 20–40 years old was 70% in Indonesia and 59% in Malaysia. There were no patients aged more than 60 in Indonesia, but there were 14% patients in Malaysia. Finally, the proportion of patients with tertiary education was 55% in Indonesia and only 28% in Malaysia.

Two key lessons were derived from this study. The first was the value of multi-country assessments of the usability of emerging digital health interventions. This approach provided key insights into perceived usability across contexts and settings in which there was variation in modes of monitoring being practiced for TB care. This provided useful comparative data and insights into possible variations across countries. A second lesson was the value of early-stage involvement of key intended users of MyTBCompanion, which was critical for identifying variations in preferences and challenges for mobile phone application development. The study highlighted that the application is well-received, usable and promising and is ready for final refinement ahead of more extensive evaluation in the context of routine care delivery.

This study represents one of the pioneering efforts to implement an integrated mHealth innovation with multi-lingual features specifically designed for low-income TB populations. The development of MyTBCompanion followed a rigorous and meticulous process at every stage, beginning with a comprehensive market survey to identify the needs and preferences of the target audience. This was followed by the iterative development of prototypes, ultimately leading to the creation of a fully functional app. Throughout this process, MyTBCompanion underwent multiple rounds of testing, including evaluations by TB experts and industry professionals, as well as field tests involving TB patients in both Malaysia and Indonesia. This thorough approach ensured that the app is not only user-friendly but also effectively addresses the unique challenges faced by its users in managing their treatment. Further, the number of patients recruited (i.e., 49) hailing from several regions is more than what has been reported in some of the literature, such as usability studies for TB (n = 12) [14] and nine [26] and breast cancer (n = 22) [27].

Nevertheless, the study is not without its limitations. A limitation of the study was the low response rates for qualitative feedback from participants, which is crucial for gaining deeper insights into their personal experiences, perceptions, and reflections. The limited feedback reduced the ability to identify specific participant-driven variables or insights that might have influenced the study’s results, hence restricting a fuller understanding of the nuances and contextual factors that are typically captured through detailed qualitative data. Future work will begin to explore which specific components of MyTBCompanion drive engagement and sustain use. The development of the underlying programme theory is a key target for future development of the intervention and a crucial element to explore within the development of any complex health intervention [28]. Factors related to non-adherence to treatment in TB patients are often patient-specific (e.g., relating to ethnicity, gender, age, cultural belief systems, comorbidities, knowledge about TB, and beliefs relating to the efficacy of medication) [29,30]. Future work to characterize different users and their expectations from the use of MyTBCompanion may develop the platform to provide more personalized, tailored support. This would also provide an opportunity to, for example, augment digital health engagement more broadly based on knowledge and experience of digital technologies. By supporting the engagement of patients with mobile phone-based applications for one condition (i.e., TB), there is scope to support skill and confidence acquisition for wider mobile phone use for health [24]. However, potential facilitators and barriers may need to be considered as part of future development. The further development and implementation of MyTBCompanion requires technological access that may not be consistent across patients and must maintain the confidence of patients in the security of their data, particularly given TB may carry social stigma in some communities [31]. A potential facilitator of further scale-up and development of MyTBCompanion is the supportive policy environment with national TB control programs that are increasingly incorporating technology to improve TB management in both Indonesia [32] and Malaysia [33]. Finally, it is to note that the current study focused on assessing the usability of MyTBCompanion, therefore, the results do not reflect TB treatment compliance. To evaluate the efficacy of any digital solution, the intended users (i.e., TB patients and healthcare professionals) must use the technology over a specific period. Since TB patients generally require at least two months of monitoring, it would be ideal for the app to be used for a minimum of two months before assessing its effectiveness in improving TB treatment compliance, as well as its impact on TB care and management. The current study aims to pursue this as the next step

## Conclusion

This study developed and assessed MyTBCompanion - an integrated A-VOT mobile app to improve TB treatment adherence in Malaysia and Indonesia. Our findings suggest an overall good agreement on the app’s usability in terms of engagement, functionality, information and aesthetics, with many patients indicating their willingness to recommend it to others, marking an encouraging milestone in the app’s development. While MyTBCompanion’s primary focus is presently centered on supporting A-VOT, our future vision involves a broader scope, which includes scaling up the app to encompass additional functionalities, such as TB testing and monitoring. The ultimate goal is to transform MyTBCompanion into a comprehensive mobile phone application that holistically addresses the diverse needs and care requirements of TB patients, ensuring their well-being through a versatile and adaptable platform.

## Supporting information

S1 Appendix MyTBCompanion features. (DOCX)

S2 Appendix Pre and post-test questionnaires. (DOCX)
