# Supplementary figures and images for: Developing and assessing MyTBCompanion – A tri-lingual integrated video observed therapy app for tuberculosis patient management in Malaysia and Indonesia
Source: PLoS One. 2025 Apr 29;20(4):e0320394. doi: 10.1371/journal.pone.0320394 (PMC12040230; doi:10.1371/journal.pone.0320394)

# PATIENT

Additional Screens – features #1 and 2 are provided in the manuscript


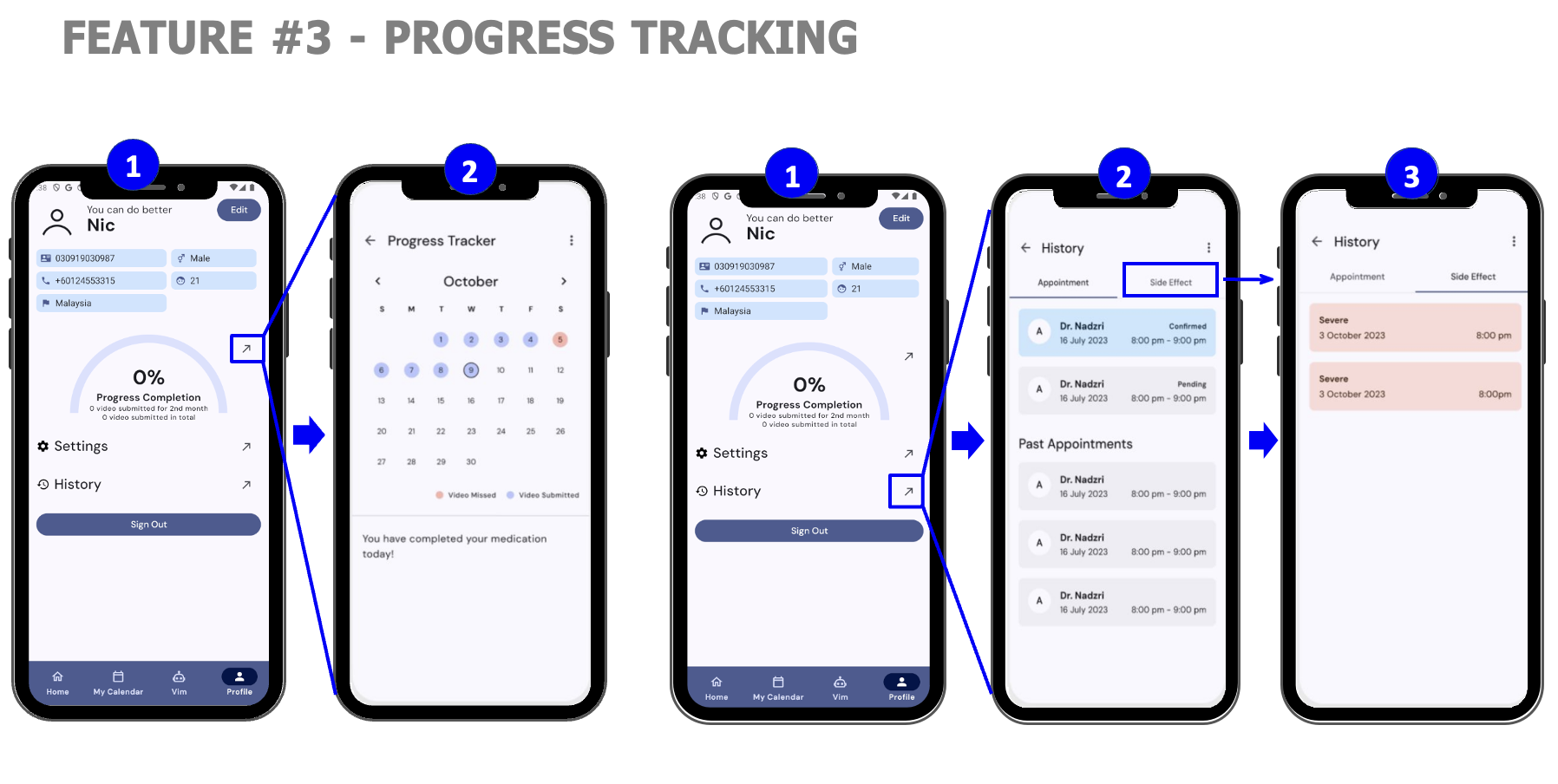


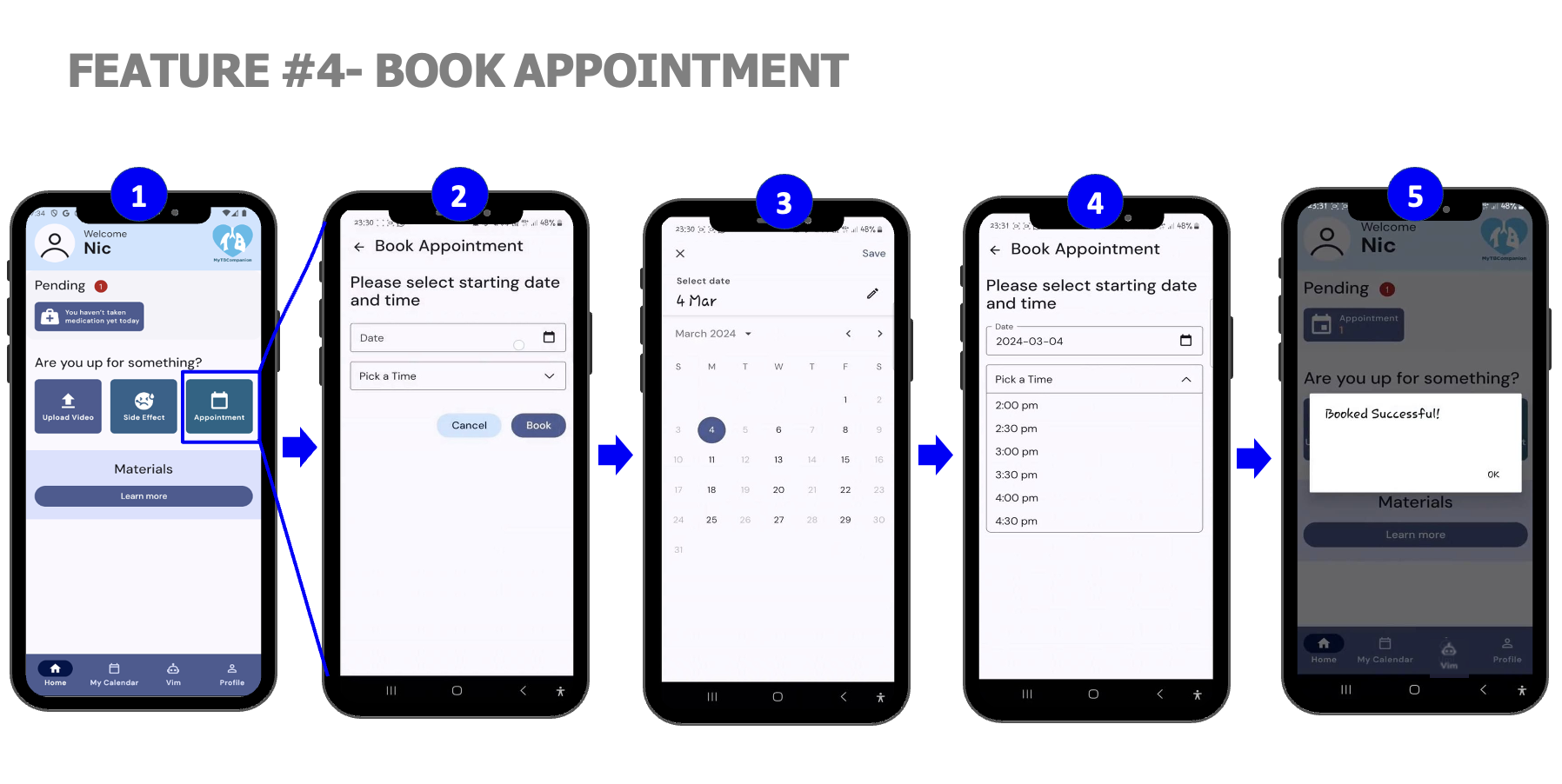


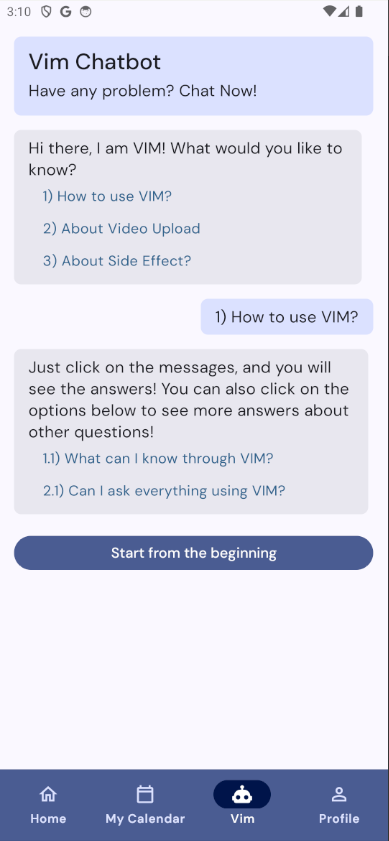

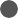

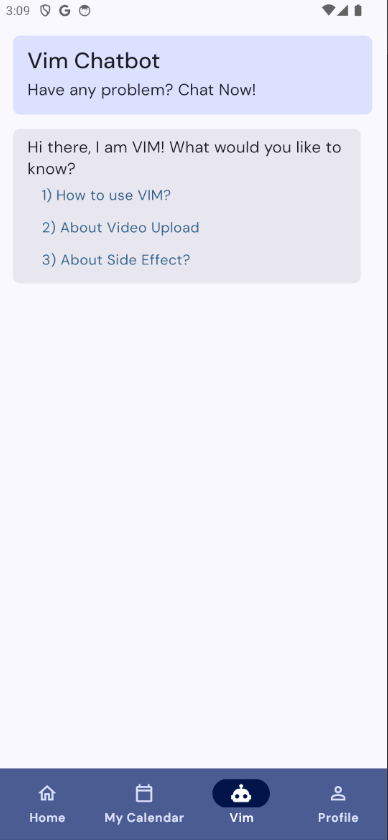

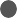

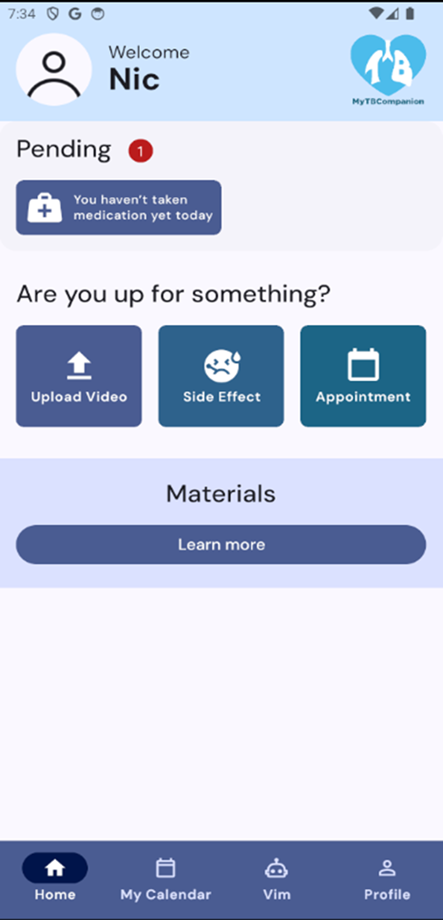

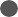

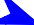


**1**

**2**

**3**

# HEALTHCARE


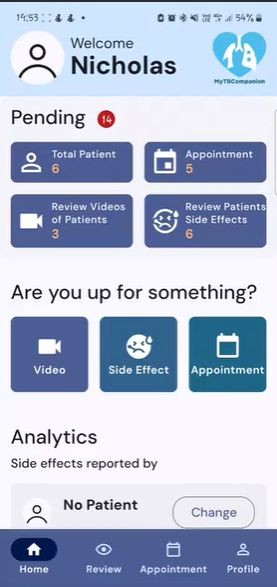

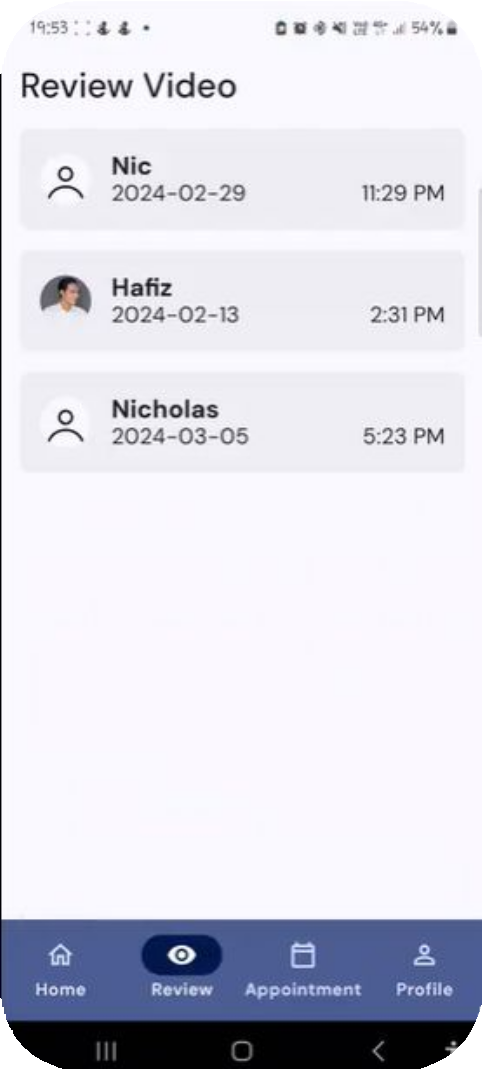

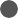

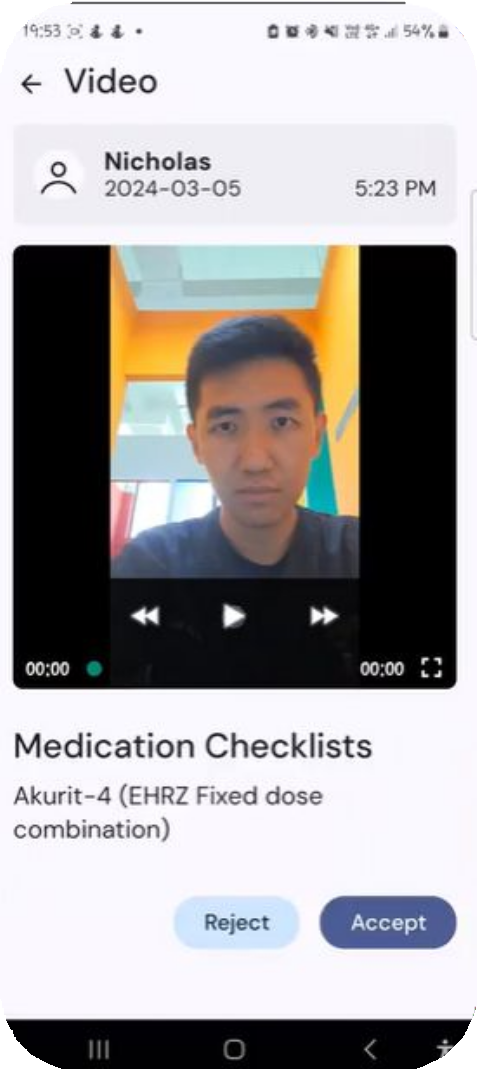

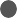

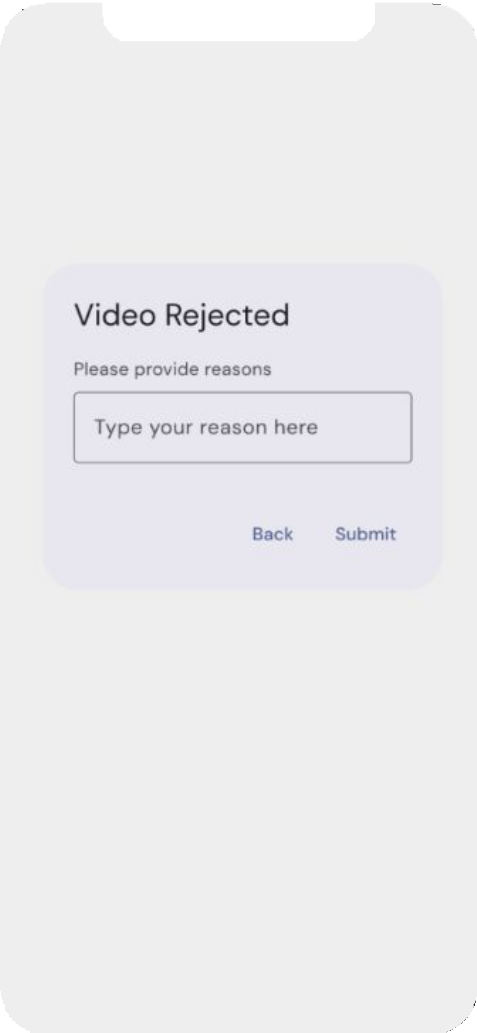

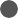


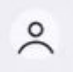
**1 2 3 4**


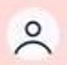

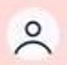

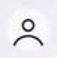


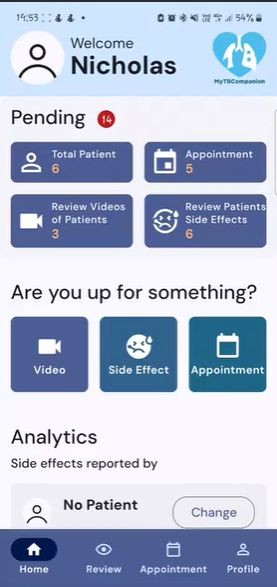

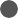

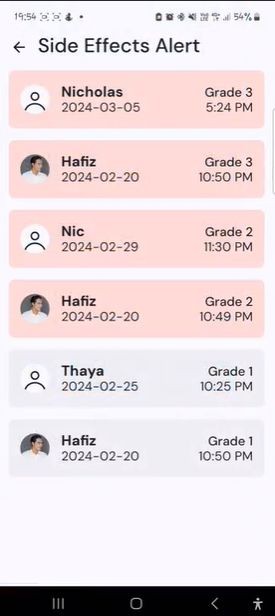

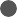

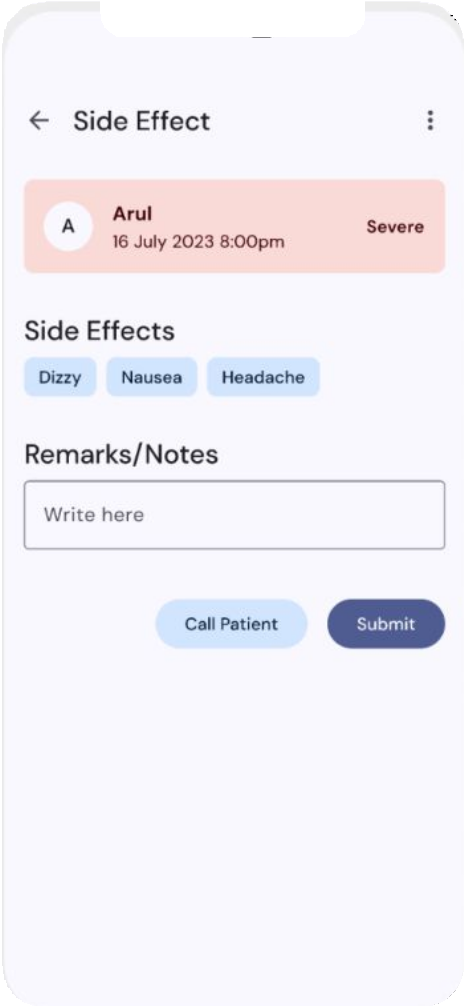

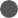


**1**

**2**

**3**


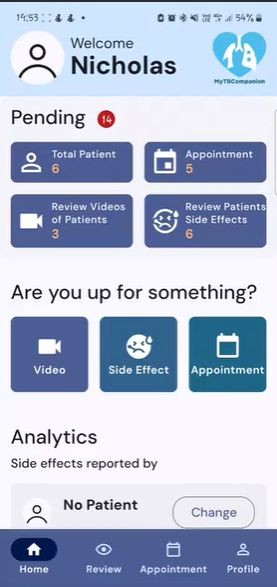

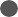

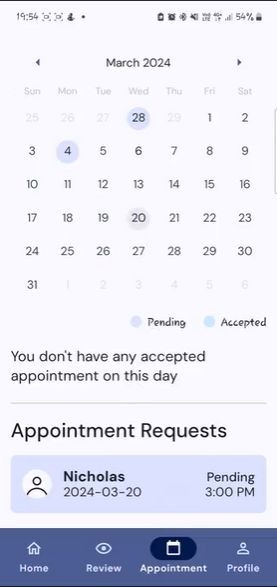

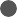

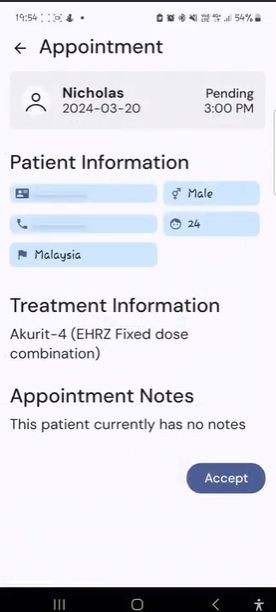

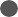

Supplement: S1 Appendix — (DOCX) [file pone.0320394.s001.docx]
