# Supplementary material for: Developing and assessing MyTBCompanion – A tri-lingual integrated video observed therapy app for tuberculosis patient management in Malaysia and Indonesia
Source: PLoS One. 2025 Apr 29;20(4):e0320394. doi: 10.1371/journal.pone.0320394 (PMC12040230; doi:10.1371/journal.pone.0320394)
Supplement: S2 Appendix — (DOCX) [file pone.0320394.s002.docx]

**Pre-test Survey**

1

***Tinjauan Pra-ujian***

This survey is to gather your opinion on the current Tuberculosis (TB) treatment strategy. Please answer the questions based on your own experience. This survey takes less than 10 minutes to complete. Your responses and details will be treated with the strictest confidentiality.

*Tinjauan ini bertujuan untuk memperoleh pendapat mengenai strategi rawatan Tuberkulosis (TB) anda kini. Sila jawab berdasarkan pengalaman anda. Tinjauan ini mengambil masa kurang daripada 10 minit untuk diselesaikan. Jawapan dan butiran anda akan dilayan dengan kerahsiaan yang paling ketat.*

Eligibility/ *Kelayakan*:

Age more than 18 years old

*Umur lebih daripada 18 belas tahun*;

Diagnosed with Tuberculosis and currently on DOTS or VOTS strategy.

*Dijangkiti dengan penyakit Tuberkulosis (TB) dan sedang menjalankan rawatan TB menerusi strategi DOTS atau VOTS.*

**Part A/ *Bahagian A*:** Demographic/*demografik*

1. Age/*Umur*: 20 – 40 41 – 60 61 and above/*61 dan ke atas*
2. Education/ *pendidikan*:

Primary/*Sekolah rendah* Secondary/*Sekolah menengah* Tertiary/*Pendidikan tertiari*

1. Experience in using mobile phone/*Pengalaman menggunakan telefon bimbit:*

< 5 years /*tahun* 6 – 15 years/*tahun* > 16 years/*tahun*

1. Do you know what Video Observed Therapy (VOT) is?

*Adakah anda tahu berkenaan Video Observed Therapy (VOT)?*

Yes/*Ya*  No/*Tidak*

1. Current treatment strategy

*Strategi Rawatan Kini*

DOTS (Jump to Part B)/*DOTS (Menerusi ke Bahagian B)*

VOTS using WhatsApp (Jump to Part C)/*VOTS menggunakan WhatsApp (Menerusi ke Bahagian C)*

VOTS using MySejahtera (Jump to Part C)/*VOTS menggunakan MySejahtera (Menerusi ke Bahagian C)*

1. How long you are on treatment?

Berapa lamakah anda sudah menjalani rawatan?

Less than one month/*Kurang daripada satu bulan*

Between 1 – 2 months/*Antara 1 – 2 bulan*

More than 2 months*/Lebih daripada 2 bulan*

**Part B:** This section is for DOTS patients.

***Bahagian B:*** *Bahagian ini adalah untuk pesakit DOTS.*

This part contains 5 statements. Please rate each of the statements below using the scale given based on your DOTS strategy.

*Bahagian ini mengandungi 5 pernyataan. Sila nilaikan setiap pernyataan di bawah menggunakan skala yang diberikan:*

1: Strongly disagree/*Sangat tidak setuju* 2: Disagree/ *Tidak setuju* 3: Neutral 4: Agree/ *Setuju* 5: Strongly agree/ *Sangat setuju*

| No | Statement/*Penyataan* | 1 | 2 | 3 | 4 | 5 |
| --- | --- | --- | --- | --- | --- | --- |
| 1 | It is easy for me to use DOTS*.*  *DOTS mudah untuk digunakan.* |  |  |  |  |  |
| 2 | It is convenient for me to use DOTS.  *DOTS sesuai untuk digunakan.* |  |  |  |  |  |
| *3* | It is effective to use DOTS.  *DOTS efektif untuk digunakan.* |  |  |  |  |  |
| 4 | I am satisfied in DOTS strategy.  *Saya berpuas hati dengan strategi DOTS.* |  |  |  |  |  |
| 5 | I am very comfortable in using DOTS.  *Saya sangat selesa menggunakan DOTS.* |  |  |  |  |  |

**Part C:** This section is for VOTS patients (Whatsapp, MySejahtera etc.)

***Bahagian C:*** *Bahagian ini adalah untuk pesakit VOTS (WhatsApp, MySejahtera dan sebagainya.)*

This part contains 10 statements. Please rate each of the statements below using the scale given based on your VOTS strategy.

*Bahagian ini mengandungi 10 pernyataan. Sila nilaikan setiap pernyataan di bawah menggunakan skala yang diberikan:*

1: Strongly disagree/*Sangat tidak setuju* 2: Disagree/ *Tidak setuju* 3: Neutral 4: Agree/ *Setuju* 5: Strongly agree/ *Sangat setuju*

| No | Statement/*Penyataan* | 1 | 2 | 3 | 4 | 5 |
| --- | --- | --- | --- | --- | --- | --- |
| **Engagement/ *penglibatan*** | | | | | | |
| 1 | Current strategy is interesting to use.  *Strategi yang digunakan sekarang menarik untuk digunakan.* |  |  |  |  |  |
| 2 | Current strategy allows customization (setting, preferences, notifications etc.  *Strategi yang digunakan sekarang membenarkan penyesuaian (tetapan, keutamaan, pemberitahuan dan lain-lain).* |  |  |  |  |  |
| *3* | Current strategy is interactive – allows user input, support feedback, notifications etc*.*  *Strategi yang digunakan sekarang bersifat interaktif – membenarkan input pengguna, maklum balas sokongan, pemberitahuan dan lain lain.* |  |  |  |  |  |
| 4 | Current strategy is suitable for TB patients.  *Strategi yang digunakan sekarang sesuai untuk pesakit TB* |  |  |  |  |  |
| **Functionality /*kefungsian*** | | | | | | |
| 5 | It is easy to learn how to use current strategy.  *Ianya mudah untuk belajar cara menggunakan strategi yang digunakan sekarang* |  |  |  |  |  |
| 6 | Screen navigations are easy, logical, and appropriate.  *Navigasi skrin adalah mudah, logik, dan sesuai* |  |  |  |  |  |
| 7 | Current strategy is easy to use.  *Strategi yang digunakan sekarang mudah digunakan* |  |  |  |  |  |
| **Aesthetics /*estetika*** | | | | | | |
| 8 | The layout (arrangement and size of buttons/icons/menus/content) is appropriate.  *Susun atur (susunan dan saiz butang/ikon/menu/kandungan) adalah sesuai.* |  |  |  |  |  |
| 9 | Graphics (buttons/icons/menus/content) are clear and of high resolution.  *Grafik (butang/ikon/menu/kandungan) adalah jelas dan mempunyai resolusi tinggi* |  |  |  |  |  |
| 10 | I like the visual interface design.  *Saya suka reka bentuk antara muka visual* |  |  |  |  |  |

**Part D - Subjective**

**Bahagian D: Subjektif**

1. Would you recommend current strategy to people who might benefit from it?

*Adakah anda akan mengesyorkan strategi yang digunakan sekarang kepada orang yang mungkin mendapat manfaat daripadanya?*

No/*tidak* Maybe/*Mungkin* Yes*/Ya*

1. What is your overall rating for Current strategy?

*Apakah rating keseluruhan anda untuk strategi yang digunakan sekarang?*

* ** *** **** *****

Please provide any suggestions or ideas to improve the current strategy

*Sila berikan sebarang cadangan atau idea untuk menambah baik strategi sekarang.*

**THANK YOU/*TERIMA KASIH***

**Post-test Survey**

2

***Tinjauan pasca-ujian***

MyTBCompanion is an integrated mobile app to improve Tuberculosis (TB) treatment adherence using Video Observed Therapy (VOT). We seek your kind assistance in helping us to provide feedback to assess the usability of MyTBCompanion.

*MyTBCompanion adalah prototaip aplikasi mudah alih bersepadu untuk meningkatkan pematuhan rawatan Tuberkulosis (TB) menggunakan VOT. Kami meminta bantuan anda dengan memberikan maklum balas kepada kita untuk menilai kebolehgunaan MyTBCompanion.*

**Instructions:** Please answer this survey after completing the Post-test and app demo/video sessions. There are three parts in this survey. Please answer all.

***Arahan****: Tolong jawab tinjauan ini selepas menyelesaikan ujian pasca-ujian dan sesi video/demonstrasi pengenalan aplikasi. Terdapat tiga bahagian dalam tinjauan ini. Tolong jawab semua.*

**Part A/*Bahagian A*:** Demographic/*demografik*

1. Which version of MyTBCompanion did you use?

*Versi MyTBCompanion manakah anda gunakan?*

Malay/Bahasa Melayu English Indonesia

**Part B*/Bahagian B*: Usability questions/*Soalan kebolehgunaan***

This part contains 17 statements. Please rate each of the statements below using the scale given:

*Bahagian ini mengandungi 17 pernyataan. Sila nilaikan setiap pernyataan di bawah menggunakan skala yang diberikan:*

1: Strongly disagree/*Sangat tidak setuju* 2: Disagree/ *Tidak setuju* 3: Neutral 4: Agree/ *Setuju* 5: Strongly agree/ *Sangat setuju*

| No | Statement/*Penyataan* | 1 | 2 | 3 | 4 | 5 |
| --- | --- | --- | --- | --- | --- | --- |
| **Engagement/*Penglibatan*** | | | | | | |
| 1 | MyTBCompanion is fun/entertaining to use.  *MTBCompanion seronok/ menghibur untuk digunakan.* |  |  |  |  |  |
| 2 | MyTBCompanion is interesting to use.  *MyTBCompanion menarik untuk digunakan.* |  |  |  |  |  |
| 3 | MyTBCompanion allows customization (setting, preferences, notifications etc.)  *MyTBCompanion membenarkan penyesuaian (tetapan, keutamaan, pemberitahuan dll.).* |  |  |  |  |  |
| *4* | MyTBCompanion is interactive – allows user input, support feedback, notifications etc*.*  *MyTBCompanion bersifat interaktif – membenarkan input pengguna, maklum balas sokongan, pemberitahuan dan sebagainya.* |  |  |  |  |  |
| 5 | MyTBCompanion is suitable for TB patients.  *MyTBCompanion sesuai untuk pesakit TB* |  |  |  |  |  |
| **Functionality /*kefungsian*** | | | | | | |
| 6 | It is easy to learn how to use MyTBCompanion (simple design)  *Mudah untuk belajar cara menggunakan MyTBCompanion (reka bentuk mudah)* |  |  |  |  |  |
| 7 | MyTBCompanion is easy to use.  *MyTBCompanion mudah digunakan* |  |  |  |  |  |
| 8 | Screen navigations are easy, logical, and appropriate.  *Navigasi skrin adalah mudah, logik dan sesuai* |  |  |  |  |  |
| 9 | Interactions between components are consistent and easy (clicking, scrolling etc.)  *Interaksi antara komponen adalah konsisten dan mudah (mengklik, menatal dan lain lain).* |  |  |  |  |  |
| 10 | MyTBCompanion works accurately without error.  *MyTBCompanion berfungsi dengan tepat dan tiada kesilapan.* |  |  |  |  |  |
| **Aesthetics/ *estetika*** | | | | | | |
| 11 | The layout (arrangement and size of buttons/icons/menus/content) is appropriate.  *Susun atur (susunan dan saiz butang/ikon/menu/kandungan) adalah sesuai* |  |  |  |  |  |
| 12 | Graphics (buttons/icons/menus/content) are clear and of high resolution.  *Grafik (butang/ikon/menu/kandungan) adalah jelas dan mempunyai resolusi tinggi* |  |  |  |  |  |
| 13 | I like the visual interface design.  *Saya suka reka bentuk antara muka visual* |  |  |  |  |  |
| **Information/ *infomasi*** | | | | | | |
| 14 | Content in the app is accurate.  *Kandungan dalam aplikasi adalah tepat.* |  |  |  |  |  |
| 15 | Content in the app is relevant to TB patients.  *Kandungan dalam aplikasi adalah relevan kepada pesakit TB.* |  |  |  |  |  |
| 16 | Content in the app including the learning materials is of good quality.  *Kandungan dalam aplikasi termasuk bahan pembelajaran adalah berkualiti.* |  |  |  |  |  |
| 17 | The app contains various visual styles – images, text and videos.  *Aplikasi ini mengandungi pelbagai gaya visual- gambar, teks dan video.* |  |  |  |  |  |

**Part C – Subjective/ *Bahagian C -Subjektif***

1. Would you recommend MyTBCompanion to people who might benefit from it?

*Adakah anda akan mengesyorkan MyTBCompanion kepada orang yang mungkin mendapat manfaat daripadanya?*

No/*tidak* Maybe/*Mungkin* Yes*/Ya*

1. Would you pay for MyTBCompanion?

*Sanggup bayar untuk MyTBCompanion?*

No/*tidak* Maybe/*Mungkin* Yes*/Ya*

1. What is your overall rating for MyTBCompanion?

*Apakah rating keseluruhan anda untuk MyTBCompanion?*

* ** *** **** *****

Please provide any suggestions or ideas to improve MyTBCompanion

*Sila berikan sebarang cadangan atau idea untuk menambah baik MyTBCompanion*

**THANK YOU/*TERIMA KASIH***
